# Insects as Food

**Published:** 1871-01

**Authors:** 


### INSECTS AS FOOD.

INTERESTING TO GOURMANDS.

Rev. Dr. Nash is publishing in *Zion's Herald* a series of articles on "Insect Life." On the delicate subject of food he says:

"Man does not refuse to use insects as a food. Even we, highly civilized as we are, do not reject the lobster, the crab, or the shrimp, which though not strictly insects, are only articulate animals, and until recently were classified with insects by our best entomologists. Now the Arab would be disgusted to see us feeding on lobster salad; yet he finds great delight in masticating a locust. In both the Indies, epicures eat the grub of the palm weevil, which is as large as your thumb; and Sir John La Forey concurs in opinion with the ancient Greeks mentioned by Ælian as esteeming roasted grub very delicious food.

"Pliny tells us that the Romans regarded the lossus—probably the larva of *Prionus Coriarius,* found in the oak, a very great delicacy. In Jamaica and in the Mauritius the grub of the *Prionus Damicornu*: which is as large as a man's finger,forms an article of food. The Mexican Indians prepare a drink from a beetle ( the *Cicindela curveta*) by macerating in water and spirits.

"Locusts are an article of food in many parts of the world. The Ethiopians were called locust eaters on this account by the Romans. The Arabs make them into bread, first grinding or pounding them and then mixing them with flour. They not unfrequently eat them boiled or stewed. The Hottentots esteem them highly, and grow fat on them. They all make their eggs into soup. Their traditions teach that they are indebted to some conjurer for the coming of the locusts. He lives a long way northward, they say, and removes a huge stone from the mouth of a deep pit so that the locusts escape and fly to them for food. The Moors of Barbary prefer them to pigeons.

"Cicadœ, according to Athenus and Aristotle were highly relished among the ancient Greeks.—Pliny says the Parthians used them freely for food. Our native Indians were fond of them as were those of New South Wales.

"The Chinese, who cannot afford to waste any edible thing, cook and eat the chrysalis of the silk worm and larva of the hawk moth. The caterpillars of butterflies are eaten by the natives of New Holland, and also the body of a butterfly called bugong.

"Ants have their places as articles of human diet. Hottentots eat them both raw and boiled. East Indians mix them with flour and convert them into a popular pastry. In India ants are used to flavor brandy.

"In Ceylon bees are used for food. In New Caledonia the people eat a large spider, (Aranea euduis), esteeming it a luxury. Reumer says he knew a young German lady who ate spiders. It is recorded that Anna Maria Schruemen ate them like nuts, and declared they were not unlike that fruit in taste. Leland, the celebrated astronomer, was equally fond of these delicacies; and Rosel knew a German who spread them on his bread like butter. Humboldt caps the climax of these edible monstrosities, assuring us that he has seen Indian children drag centipedes, eighteen inches long and more than half an inch broad, from their holes and devour them.

"While these curious facts illustrate the adage that there is no accounting for tastes, they also show that insects are useful as food for man, and in great extremities he might be saved from destruction by placing them among his articles of diet. But I have written enough on the uses of insects—enough to show that the Great Architect of nature did not create these curious little animals in such vast numbers without a purpose. Small as they are, and contemptible as they appear, their countless numbers and varied powers to do both good and evil, constitute them one of the most important forces in the economy of nature. As already intimated, by merely destroying a few classes of insect-fauna and thereby permitting the others to multiply indefinitely, the Almighty could bring about the entire destruction of the human race in a surprisingly brief period of time."

—A dentist in Philadelphia has traced out the career of 1,000 dentists, with the following result:—163 died before reaching 35 years; 643 attained fair success; 57 made fortunes; 27 died from intemperence and other vices; 95 failed entirely; and 3 committed suicide.

—A man in South Hadley, who has just got out of a lawsuit, wants to obtain a large framed picture of a cow, with one client at the head and the other at the tail, pulling, and the lawyers meanwhile quietly milking.

### THE FIDGETS.

The best definition of fidgets we ever read " was irritability of locomotion." An eminent physician, who thus defined it, in a humorous episode attached to a more serious dissertation, peculiar to females, especially those who are called "your mighty good kind of women." It is the result of two passions, hope and fear; and a mind continually vibrating between these, produces the disease. The whole pleasure of this kind of women is bustle. They plan their work, neither for the benefit of the house, nor of those who do the labor, but purely to divert their minds, to keep everybody in motion. They hate to see anybody in the house enjoying a moment's leisure, for idleness is, in their opinion, a greater sin than vice itself, and all leisure is idleness. But the fidgets is a disease which is not confined to women. The poor sailors speak of what they call "fidgety captains," who, after all necessary tasks are done, will actually put things into mischevious disorder for the sake of keeping constantly at work. The only cure for the fidgets is said to be hard labor constantly pursued. When the patients are wearied with toil, they have no more "irritability of locomotion," and prefer taking their rest to fatiguing themselves still more by driving others to their tasks.

POISONOUS EFFECTS OF ORANGE PEEL.—Many years ago, says Dr. Gibbons, two little girls, sisters, four and six years of age, were seized with violent inflammation of the bowels from swallowing the rind of the orange. One of them died in convulsions, and the other had a narrow escape. Quite recently, a child something over a year old was attacked with violent dysenteric symptoms, for which no cause could be assigned. The attack came on during the passage of the family on the steamer from San Diego. The symptoms were identical with those which had previously been noticed to arise from poisoning by orange peel; and on inquiry, we were informed that it had been playing with an orange and nibbling at it just before the attack of disease. The discharges from the bowels were frequent and painful, and consisted of blood and mucus. After a week of severe enteric inflammation, the child died. We have no doubt that the disease was brought on by the rind of the orange. Though but a small quantity must have been swallowed, yet a very small quantity of such an indigestible and irritating substance will often produce the most serious consequences.

